# Antimalarial properties of crude extracts of seeds of *Brucea antidysenterica* and leaves of *Ocimum lamiifolium*

**DOI:** 10.1186/s12906-016-1098-9

**Published:** 2016-04-14

**Authors:** Atetetgeb Kefe, Mirutse Giday, Hassen Mamo, Berhanu Erko

**Affiliations:** Department of Microbial, Cellular and Molecular Biology, College of Natural Sciences, Addis Ababa University, P.O.Box 1176, Addis Ababa, Ethiopia; Aklilu Lemma Institute of Pathobiology, Addis Ababa University, P. O. Box 1176, Addis Ababa, Ethiopia

**Keywords:** Malaria, *Plasmodium berghei*, *Brucea antidysenterica*, *Ocimum lamiifolium*, Ethiopia

## Abstract

**Background:**

The search for new antimalarial drugs has become increasingly urgent due to plasmodial resistance to existing drugs. As part of this global effort, the present study aimed at evaluating the antimalarial activity of two traditionally used medicinal plants against the disease.

**Methods:**

Acute toxicity and four-day suppressive effects of aqueous, methanol and chloroform extracts of the seed and leaf of *Brucea antidysenterica* and *Ocimum lamiifolium,* respectively, were investigated in Swiss albino mice using *Plasmodium berghei* using standard procedures.

**Results:**

Methanol extract of the leaves of *O. lamiifolium* did not exhibit any sign of acute toxicity up to the dose of 2000 mg/kg body weight. However, all mice provided with seeds of *B. antidesenterica* at a dose of 2000 mg/kg body died within 24 h. The aqueous, methanol and chloroform crude extracts of *B. antidesenterica* significantly (*p* < 0.05) inhibited parasitaemia in a dose-dependent manner and prevented body weight loss at doses of 200, 400 and 600 mg/kg body weight. In addition, the extracts prolonged the mean survival time of *P. berghei*-infected mice compared to the non-treated control. However, it did not prevent reduction in packed cell volume except the chloroform extract in three doses and methanol extract at 200 mg/kg and 400 mg/kg. Extracts from *O. lamiifolium* also exhibited significant (*p* < 0.05) antiplasmodial activities. The extracts did not prevent body weight loss and PCV reduction, especially in chloroform. The highest suppression was recorded from aqueous crude extract of *O. lamiifolium* with 35.53 % in the dose of 600 mg/kg. On the other hand, a similar higher suppression was found in both methanol and chloroform of crude extracts of *B. antidesenterica* with 47.70 %, 46.44 % of chemosuppression, respectively, in its highest dose tested.

**Conclusion:**

Crude aqueous, methanol and chloroform extracts of the two medicinal plants possess acceptable antimalarial effects. However, further investigation should be pursued on toxicity study and to isolate the bioactive components responsible for the observed antimalarial action of the plants.

## Background

Malaria has been a great challenge to humanity since time immemorial. An estimated 300–500 million people are affected by malaria throughout the world annually [1]. This same world health organization (WHO) source indicates that 95 % of malaria-related deaths occur in sub-Saharan Africa, with children younger than five years of age and pregnant women being the most severely affected. In recent years, the burden of malaria has fallen in many parts of Africa following the introduction of more effective treatments and the scale-up of long-lasting insecticidal nets (LLIN) use [2]. But, vector mosquito resistance to insecticides recommended for current use [3] coupled with emergence of drug-resistant parasite strains to the latest antimalarials in use [4–6] demand for new drugs with new modes of action.

Plants have been used in the traditional treatment of malaria for years in the past and present in various parts of the world [7] apart from being source of various antimalarial drugs [8]. In Ethiopia it is estimated that about 90 % of the population is dependent on traditional medicine, essentially plants [9].

*Ocimum lamiifloium* Hochst. ex Benth and *Brucea antidysenterica* Mill are among the most common traditional medicinal plants used in Ethiopia. *O. lamiifolium* belongs to the family Lamiaceae. It is traditionally used to relieve pain, wound, fever, malaria and inflammatory disorders in Ethiopia [10]. It is also used for treatment of intestinal disorders, eye disease and cough [11]. *Brucea antidysenterica* (Simaroubaceae) is similarly used in traditional medicine for multiple purposes. Different parts of the plant are widely used against malaria, helminthic infections, fever, dysentery and other disorders [11, 12]. Despite its wide application in the traditional healthcare domain only few organized and thorough scientific investigations have been undertaken to evaluate the safety and efficacy of Ethiopian traditional medicinal plants. In vivo and in vitro studies conducted on fruit extracts of *Brucea javanica*, a species closely related to *Brucea antidysenterica,* demonstrated antiplasmodial activity of the plant attributable to quassinoid constituents [13]. Study also shows that the organic leaf extract of *Ocimum gratissimum*, a species found under the genus where *Ocimum lamiifolium* belongs, suppressed parasitaemia of *Plasmodium berghei* in mice by 88.07 % at a dose of 100 mg/Kg body weight [14]. Essential oil of *Ocimum gratissimum* demonstrated significant antimalarial activities in the four-day suppressive in vivo test in mice [15]. In vitro test conducted on three quinones isolated from *Ocimum basilicum*, another related species, showed antiplamodial activity with an IC50 value of below 1 μg/ml [16]. The present study, therefore, aimed at assessing the antimalarial activity of the crude extracts of the seed of *B. antidysenterica* and leaf of *O. lamifolium* against *P. berghei* in Swiss albino mice.

## Methods

### Plant materials and identification

The plant samples were collected from Endeget Yfotie area (Lummamie District) in Amhara Regional State, 260 km northwest of Addis Ababa, Ethiopia, in October, 2013. The plants were identified by Dr Mirutse Giday (second author) at Aklilu Lemma Institute of Pathobiology, Addis Ababa University, and voucher specimens were deposited in the National Herbarium of Addis Ababa University with voucher number AK01 (*O. lamiifolium*) and AK02 (*B. antidysenterica*).

### Preparation of crude plant extracts

The collected plant parts were air-dried at room temperature under shade, without exposure to sun light, and ground into powder using an electric mill (IEC, 158 VDE 066, Germany). The crude extracts were prepared by a cold maceration technique [17]. The powders of plant parts (90 gm of each part) were separately mixed in 900 ml of distilled water, chloroform and methanol in Erlenmeyer flasks and placed on a platform shaker (GFL, model 3020, Germany) of 120 revolutions per minute (rpm) for 72 h at room temperature. The mixtures were first filtered using muslin gauze and then passed through Whatman grade № 1 filter paper. The methanol and chloroform extracts were concentrated in a rotary evaporator (Buchi type TRE121, Switzerland) at a temperature of 45 °C and dried in water bath at 45 °C. The water extracts were frozen in a refrigerator overnight and dried in a lyophilizer (CHRIST, 3660 Osterode/harz, France) to get a freeze-dried product.

### Experimental animals and *Plasmodium* parasite inoculation

Swiss albino mice (6-8weeks) of both sexes weighing 27–36 g and chloroquine (CQ)-sensitive *P. berghei* strain maintained in the animal house of the Department of Microbial, Cellular and Molecular Biology, Addis Ababa University, were used for the experiment. The mice were housed in cages under standard conditions and all had free access to food (mice pellet) and water *ad libitum* throughout the experimental period. Good hygiene was maintained by constant cleaning and removal of feces and spilled food from cages daily. The parasite was subsequently maintained in the laboratory by serial blood passage from infected mice to the non-infected ones on a weekly basis. Blood samples taken from donor mouse with growing parasitaemia of 30–40 % were diluted with normal saline so that each 0.2 ml of blood contained 1×10^7^ infected erythrocytes, the standard inocula [18]. Each mouse was intraperitoneally inoculated with 0.2 ml of diluted blood.

### In vivo acute toxicity test of the crude plant extracts

The methanol extract of the two medicinal plants were evaluated for their acute toxicity in non-infected female mice. The mice were starved for 3–4 h before the experiment with only water allowed and 1–2 h after the administration of the extracts. In the evaluation of each extract, 20 mice were randomly divided into four groups of 5 mice per cage and were given orally increasing doses of 500, 1000 and 2000 mg/kg body weight of the plant extracts. The mice in the control group received 0.2 ml of respective vehicle of each extract (3 % Tween 80). Then, the mice were observed continuously for 1 h, intermittently for 4 h and a period of 24 h for gross behavioral changes such as rigidity, sleep, mortality and other signs of acute toxicity manifestations and were followed for 14 days [19].

### In vivo evaluation of the antimalarial activity of plant extracts

For an in vivo evaluation of each extract, the standard four-day suppressive method was used [4]. Male mice were infected by giving inocula of about 1×10^7^ parasites. The infected mice were randomly divided into 11 test groups (5 mice in each group); nine test groups and two control groups (each for CQ as a standard drug and 3 % tween 80 as a negative control) for each plant. The test extracts were prepared in three different doses; 200 mg/kg, 400 mg/kg and 600 mg/kg and CQ at 25 mg/kg in a volume of 0.2 ml and vehicles at 1.4 ml/mouse in a volume of 0.2 ml. All the extracts and the drug were given through intragastric route by using standard intragastric tube to ensure safe ingestion of the extracts and the drug. Treatment was started after 3 h of infection on day 0 and then continuing daily as a single dose for four days (*i.e*. from day 0 to day 3). On the fifth day (D_4_), blood sample was collected from tail snip of each mouse. Thin smear was fixed with methanol and stained with 10 % Giemsa solution.

The stained slides were examined under light microscope with an oil immersion objective of 100× magnification power to evaluate the percent suppression of each extract with respect to the control groups. Respective percent parasitaemia and percentage suppression were calculated using the formula given below [20, 21].

$$ \%\  Suppression=\frac{Parasitaemia\  in\  negative\  control- Parasitaemia\  in\  study\  group\ }{Parasitiaemia\  in\  negative\  control} \times 100 $$

$$ \%\  Parasitaemia=\frac{Number\  of\  infected\  RBCs\ }{Total\  No\  of\  RBCs\  examined}\kern0.5em \times 100 $$

#### Determination of packed cell volume

Packed cell volume (PCV) of each mouse was measured before infection (D_0_) and on D4 after infection. For this purpose, blood was collected from the tail of each mouse in heparinized microhaematocrit capillary tubes up to 3/4^th^ of its length. The tubes were sealed by crystal seal and placed in a microhematocrit centrifuge (Hettich haematokrit) with the sealed ends outwards. The blood was centrifuged at 12,000 rpm for 5 min. The volume of the total blood and the volume of erythrocytes were measured and PCV was calculated as given below [22]:

$$ PCV=\frac{Volume\  of\  erythrocytes\  in\ a\  given\  volume\  of\  blood\kern0.5em }{Total\  blood\  volume}\times 100\% $$

#### Determination of body weight

The body weight of each mouse in all the groups was measured on D0 and on D4 in case of treatment to evaluate the effectiveness of the extracts in preventing loss of body weight due to the parasite. Each mouse was weighted before and after the different doses using sensitive digital weighing balance (Scientech balance).

#### Determination of mean survival time

Mortality of both the treatment and control groups were monitored and recorded daily for each mouse for one month. The mean survival time (MST) for each group was calculated using the following formula as described below [22].

$$ MST=\frac{Sum\  of\  survival\  time\ (days)\  of\  all\  mice\  in\ a\  group}{Total\  number\  of\  mice\  in\  that\  group} $$

### Data analysis

Data were presented as mean plus or minus standard error of the mean (M ± SEM). Statistical significance was determined by one-way analysis of variance (ANOVA) and Post hoc’s Scheffe test using statistical package for social sciences (SPSS) version 17 software (Chicago, IL, USA). Student's paired t – test was used to compare parameters within groups. For all the data obtained, statistical significance was set at *p* < 0.05.

## Results

### Extraction yields of plant materials

For seeds of *Brucea antidysenterica*, the highest yield was obtained for their methanol extract (8.69 g), followed by that for aqueous (5.64 g) and chloroform (4.79 g) extracts. For leaves of *Ocimum lamiifolium*, the highest yield was obtained for their methanol extract (7.79 g), followed by that for aqueous (6.20 g) and chloroform (5.21 g) extracts. However, there was no significant difference (*p* > 0.05) in yield among the different extracts.

### In vivo acute toxicity test

The methanol extracts of the leaves of *O. lamiifolium* administered orally in a single dose of up to 2000 mg/kg body weight, showed no lethal effect within 24 h of observation. Gross physical and behavioral observation of the experimental mice revealed no visible signs of acute toxicity such as urination, hair erection, lacrimation, and reduction in feeding activity. In general, the mice were physically active. However, in case of the aqueous, chloroform and methanol extracts of the *B. antidysenterica* extract, lack of visible signs of acute toxicity and mice fatality were observed in the dose of 500 mg/kg body weight. At the level of 1000 mg/kg body weight, hair erection and sleepy activities were observed within 4 h although the mice became active from that time onwards. But, in the dose of 2000 mg/kg body weight all mice were dead within 24 h.

### In vivo evaluation of the antimalarial activity of plant extracts

#### Effect of crude extracts on PCV and body weight

The result showed a significant (*p* < 0.05) reduction in packed cell volume (PCV) values between D0 (inoculation day) and D4 (5 days after D0) in mice treated with chloroform and methnol extracts of the leaves of *O. lamiifolium* at all the three dose levels (200 mg/kg, 400 mg/kg for chloroform and 600 mg/kg). Whereas, mice treated with aqueous extract did not show significant (*p* > 0.05) PCV reduction at all the three dose levels. There was significant (*P* < 0.05) change between D0 and D4 in body weight of mice treated with chloroform extracts at the three dose levels. The aqueous and methanol extracts treated mice had shown no significant (*P* > 0.05) deviation in body weight between D4 and D0 (Table 1).

**Table 1:** Effect of aqueous, methanol and chloroform crude extracts of leaves *Ocimum lamiifolium* on body weight and PCV of *P. berghei* infected mice

Extracts/treatments	Dose (mg/kg)	PCV		Body weight	
		D0	D-4	D0	D-4
NC	0	52.80 ± 0.48	40.58 ± 0.64^b^	27.90 ± 0.64	23.98 ± 1.46
dH_2_O	200	53.59 ± 2.88	44.75 ± 0. 96^a^	27.76 ± 0.20	26.88 ± 0.25^a^
	400	55.09 ± 1.01	47.49 ± 2.22^a^	28.12 ± 0.13	29.56 ± 0.32^a^
	600	51.98 ± 0.99	50.38 ± 0.52^a^	26.88 ± 0.15	27.20 ± 0.55^a^
CH_3_OH	200	54.66 ± 1.15	42.67 ± 0.67^b^	26.50 ± 1.41	26.29 ± 2.53^a^
	400	51.69 ± 1.11	40.13 ± 1.84^b^	27.56 ± 1.41	26.22 ± 1.40^a^
	600	53.98 ± 0.99	56.09 ± 0.44^b^	29.10 ± 2.58	30.09 ± 2.16^a^
CHCl_3_	200	56.94 ± 0.50	46.36 ± 0.6^b^	29.38 ± 2.39	24.36 ± 1.44^b^
	400	54.14 ± 1.56	42.30 ± 0.87^b^	33.70 ± 8.76	28.23 ± 6.90^b^
	600	51.68 ± 0.74	43.23 ± 0.66^b^	28.66 ± 2.55	25.00 ± 0.51^b^
CQ	25	54.73 ± 0.27	53.37 ± 0.21^a^	28.80 ± 0.23	30.97 ± 0.92^b^

Key: Values are presented as mean ± SEM; *n* = 5; *PCV* packed cell volume, *NC* negative control (3 % tween 80), *dH2O* distilled water, *CH*_*3*_ *OH* methanol, *CHCl*_*3*_ chloroform, *CQ* chloroquine (positive control); ^a^ = the difference between day 0 and day 4 is not significant (*P* > 0.05); ^b^ = the difference between day 0 and day 4 is significant (*P* < 0.05)

Extracts of the seeds of *B. antidysenterica* have significantly (*P* < 0.05) prevented loss of body weight between D0 and D4 in mice treated with methanol extract at all the three dose levels (200 mg/kg, 400 mg/kg and 600 mg/kg) Mice treated with chloroform extract significantly (*p* < 0.05) lost body weight only at two dose levels (200 mg/kg, 400 mg/kg) and those treated with aqueous extract demonstrated significant (*P* < 0.05) body weight loss only at the dose level of 600 mg/kg. Significant (*P* < 0.05) reduction in PCV was observed at all the three dose levels in mice treated with aqueous extract. Mice treated with methanol extract showed significant (*P* < 0.05) reduction in PCV only at the dose level of 600 mg/kg. However, no significant (*P* > 0.05) loss in PCV was shown between D0 and D4 in mice treated with chloroform extract at all the three dose levels (Table 2).

**Table 2:** Effect of aqueous, methanol and chloroform crude extracts of seeds of *Brucea antidysentmerica* on body weight and PCV of *P. berghei* infected mice

Extracts/treatments	Dose (mg/kg)	PCV		Body weight	
		D0	D-4	D0	D-4
NC		53.37 ± 0.75	46.67 ± 1.79^b^	28.70 ± 3.24	27.60 ± 3.88
dH_2_O	200	56.99 ± 2.02	45.10 ± 0.65^b^	32.25 ± 1.22	29.75 ± 0.09^a^
	400	56.84 ± 2.83	43.53 ± 5.08^b^	31.50 ± 0.79	30.24 ± 0.52^a^
	600	55.74 ± 1.00	48.62 ± 1.93^b^	25.80 ± 0.89	31.10 ± 1.37^b^
CH_3_OH	200	50.78 ± 0.96	51.29 ± 0.29^a^	28.56 ± 1.41	31.22 ± 1.40^b^
	400	53.19 ± 1.09	53.26 ± 2.20^a^	34.10 ± 2.58	36.09 ± 2.16^b^
	600	56.62 ± 1.90	50.40 ± 3.55^b^	31.50 ± 2.30	33.35 ± 1.86^b^
CHCl_3_	200	55.67 ± 1.38	52.89 ± 1.30^a^	30.95 ± 2.25	32.36 ± 0.28^b^
	400	52.09 ± 2.83	53.09 ± 0.95^a^	29.6 ± 1.83	31.25 ± 0.44^b^
	600	51.8 ± 1.59	51.27 ± 1.95^a^	29.5 ± 1.41	29.39 ± 0.5^a^
CQ	25	52.27 ± 0.74	51.82 ± 0.82^a^	30.54 ± 0.37	33 ± 0.37^b^

Key: Values are presented as mean ± SEM; *n* = 5; *PCV* packed cell volume, *NC* negative control (3 % tween 80), *dH2O* distilled water, *CH*_*3*_ *OH* methanol, *CHCl*_*3*_ chloroform, *CQ* chloroquine (positive control); ^a^ = the difference between day 0 and day 4 is not significant (*P* > 0.05); ^b^ = the difference between day 0 and day 4 is significant (*P* < 0.05)

#### Effect of crude extracts on parasitaemia and mean survival time

In the four day suppressive test, all the extracts resulted in reduction of parasitaemia level significantly (*P* < 0.05) as compared to the negative control for both plants. However, the extracts did not clear the parasite completely on D4 as opposed to the positive control group (treated with 25 mg/kg CQ) which had no detectable parasitaemia on the same post infection day.

The crude aqueous, methanol and chloroform extracts of *O. lamiifolium* significantly suppressed the parasitaemia. The highest suppression was obtained for methanol (24.95, 24.57 and 26.06 %) and aqueous (22.16, 26.76 and 35.53 %) extracts treated mice at the dose levels of 200 mg/kg, 400 mg/kg and 600 mg/kg, respectively. The extracts also prolonged the mean survival time of infected mice compared to the non-treated ones (Table 3).

**Table 3:** Antimalarial activities of aqueous, methanol and chloroform extracts of leaves of *O. lamiifolium* and mean survival time of *P. berghie* infected mice

Extraction solvents	Dose (mg/kg)	Extract antimalarial activity on D4 post-infection		Mean survival time (days)
		% Parasitaemia ± SEM	% suppression	
dH_2_O	200	35.68 ± 1.42	22.16	10.95 ± 1.04^*^
	400	33.57 ± 2.81	26.76	11.65 ± 0.65^*^
	600	29.55 ± 0.89	35.53	12.50 ± 0.41^*^
CH_3_OH	200	33.83 ± 3.43	24.95	10.00 ± 1.70^*^
	400	34.00 ± 5.92	24.57	12.80 ± 0.76^*^
	600	33.33 ± 11.69	26.06	11.00 ± 0.80^*^
CHCL_3_	200	38.00 ± 3.88	15.70	9.40 ± .67^*^
	400	36.42 ± 2.01	19.21	9.80 ± 0.67^*^
	600	35.75 ± 3.37	20.69	9.60 ± 1.51
Control	CQ (25 mg)	0.00 ± 0.00	100.00	ND
	T80	45.84 ± 2.11	0.00	7.00 ± 0.50

Key: Values are presented as M ± SEM; *n* = 5; T80 = negative control (3 % tween 80); *CQ* positive control (chloroquine), *ND* no death within the follow-up interval (30-days), *dH*_*2*_ *O* distilled water, *CH*_*3*_ *OH* methanol, *CHCl*_*3*_ chloroform, *CQ* chloroquine (positive control); ^*^ = values are significantly different (*p* < 0.05) from that of the negative control

In the same way, the aqueous, methanol and chloroform extracts of *B. antdysenterica* showed significant parasite suppression in all the doses administered and they exhibited significant suppression of the parasitaemia at a higher dose (600 mg/kg). The highest suppression was obtained for methanol (38.08, 43.33 and 46.44 %) and chloroform (31.23, 43.23 and 47.70 %) extracts treated mice at the dose levels of 200 mg/kg, 400 mg/kg and 600 mg/kg, respectively. The mean survival time (MST) of mice for a given extract was also longer compared to those in the negative control (Table 4).

**Table 4:** Antimalarial activities of aqueous, methanol and chloroform extracts of seeds of *B. antidysenterica* and mean survival time of *P. berghie* infected mice

Extraction solvents	*B. antidysenterica*	Extract antimalarial activity on D4 post-infection		Mean survival time (days)
	Dose (mg/kg)	% Parasitaemia ± SEM	% suppression	
dH_2_O	200	39.69 ± 1.10	24.05	9.25 ± 0.69^*^
	400	32.19 ± 0.62	38.40	10 ± 00.54^*^
	600	28.39 ± 1.68	45.68	12.50 ± 0.41^*^
CH_3_OH	200	34.36 ± 0.86	38.08	12.90 ± 1.04
	400	28.57 ± 1.31	43.33	14.80 ± 1.20^*^
	600	27.99 ± 0.89	46.44	15.00 ± 1.40^*^
CHCl_3_	200	35.94 ± 0.86	31.23	12.00 ± 1.70^*^
	400	29.67 ± 1.31	43.23	13.00 ± 0.80^*^
	600	27.33 ± 0.89	47.70	15.50 ± 0.41^*^
Controls	QC (25 mg)	0.00 ± 0.00	100.00	ND
	T80	52.26 ± 1.50	0.00	7.00 ± 0.50

Key: Values are presented as M ± SEM; *n* = 5; T80 = negative control (3 % tween 80); *CQ* positive control (chloroquine), *ND* no death within the follow-up interval (30-days), *dH*_*2*_ *O* distilled water, *CH*_*3*_ *OH* methanol, *CHCl*_*3*_ chloroform, *CQ* chloroquine (positive control); ^*^ = values are significantly different (*p* < 0.05) from that of the negative control

## Discussion

The methanol extracts of the two plants produced the highest yield and this could be due to high concentration of compounds in the plants that better dissolve in methanol. The highest yield of methanol extracts could also be attributed to relative ability of methanol to penetrate cellular membranes to extract ingredients in cells. The leaf extracts of *Ocimum lamiifolium* did not cause mortality or any sign of behavioral change that is indicative of acute toxicity in the experimental mice after oral administration of extract (500 to 2000 mg/kg body weight). Therefore, the plant can be considered safe according to the Organization for Economic Cooperation and Development (OECD) guideline which recommends a maximum dose of 2000 mg/kg for acute toxicity [19]. This could justify the common use of the plant in Ethiopian traditional medicine to treat malaria and other ailments.

In case of methanol extract of the seeds of *Brucea antidysenterica*, no visible signs of acute toxicity and death of the mice were observed at a dose of 500 mg/kg body weight while at the level of 1000 mg/kg body weight hair erection and sleepy activities were observed but no death was recorded. Meanwhile, at the dose of 2000 mg/kg body weight all mice were dead. This indicates that the lethal dose (LD_50_), a concentration of a chemical that kills 50 % of the test animals, of *B. antidysenterica* methanolic extract is less than 2000 mg/kg body weight.

All the three crude extracts (aqueous, methanol and chloroform) of *B. antidysentrica* and aqueous and methanol extracts of *O. lamiifolium* prevented weight loss. However, chloroform extract of *O. lamiifilum* did not prevent weight loss which could be attributed to the presence of compounds that have effect on appetite.

The PCV values of *P. berghei* infected mice that were treated with the extracts of both plants were reduced as a result of malaria infection. The mean survival time of mice treated with all the extracts of *B. antidysenterica* was longer compared to the respective negative control, confirming that the crude extracts of the study plant suppressed *P. berghei* and probably reduced the overall pathogenic effect of the parasite in the study mice. However, mice treated with *O. lamiifolium* extract lived shorter. This may be due to the suppressive effects of the plant. This suggests that the crude extracts of *B. antidysenterica* were relatively more protective than that of *O. lamiifolium*. It may be because of the relatively shorter half life of *O. lamiifolium* [23].

According to Krettli *et al*. [24] a compound is considered active when reduction in parasitaemia is ≥ 30 %. In this study, all test groups treated with methanol, aqueous and chloroform crude extracts of *B. antidesenterica* induced more than 30 % suppression including the lower doses except the 200 mg/kg of the aqueous extract. Similar studies on other plant species such as *Acacia nilotica* [25], *Clerodendrum myricoides, Dodonea angustifolia* and *Aloe debrana* [26] and *A. africanus* [27] reported significant parasitaemia suppression.

A remarkable suppression suggests the potency of the seed extracts of *B. antidyesenterica* against malaria. The antimalarial activity of the plant might be attributed to the presence of bioactive compounds such as quassinoid and canthin alkaloids [28]. However, the active compound(s) responsible for the observed activity need to be identified in future studies.

On the other hand, the lower doses (200 mg/kg and 400 mg/kg) of the methanol and aqueous extracts of *O. lamiifolium* may be considered to have lower antimalarial activities. This could be due to the presence of active compounds at low levels in natural products whose activity may not be detected at lower doses [24].

The difference between the antimalarial activities of extracts of both plants in all the three solvents may indicate differences in the level and composition of active compounds [29]. The difference may also be due to differences between the solvents used for the extraction of the plant materials as the secondary metabolites/compounds could be different.

The observed low antimalarial activity of plant extracts tested could be partly explained by the fact that many antimalarial traditional medicinal plants may lack direct antiplasmodial activity to cure the disease but their beneficial role could be in their antipyretic, analgestic and immune stimulatory effect as demonstrated in other studies [7, 10]. Furthermore, in traditional medicinal practice, the healers are known to prescribe concoctions made from different species of plants that might have high synergetic effects. According to Abdu *et al*. [30], biological actions can be due to these components in complicated concert of synergistic or antagonistic activities. Also, the effectiveness of a given extract can also be influenced by the rate of gastrointestinal uptake and the half life in plasma metabolism of the active compounds [23].

The extracts of the two plant parts did not totally clear the parasitaemia up to the highest tested doses. Complete clearance of parasitaemia may be achieved within the toxic range of the extracts. These findings provide information on the antimalarial effect of the plant extracts tested and warrant carrying out of chronic toxicity studies.

The limitation of this study includes failure for not conducting additional antimalarial tests using curative and prophylactic methods.

## Conclusion

This study has shown that aqueous, methanol and chloroform extracts of seeds of *B. antiydesenterica* and leaves *O. lamiifolium* exhibited a reasonable antiplasmodial activity. While *O. lamiifolium* was safe at the highest tested dose of the extracts, *B. antidysenterica* showed toxicity at 2000 mg/kg body weight. Further studies are recommended on antiplasmodial activities as well as safety profiles of the plants.

### Ethical consideration

The study obtained ethical clearance from the Institutional Ethics Review Board of College of Natural Sciences, Addis Ababa University (No: GSR/1559/05). The mice were treated as per the guidelines on the care and use of animals for scientific purposes.

### Availability of data and materials

Specimens of the two tested antimalarial plants were deposited at the National Herbarium of the Addis Ababa University with voucher numbers AK01 assigned for *Ocimum lamiifolium* and AK02 for *Brucea antidysenterica*. Data related to antimalarial *in vivo* tests of the two plants were deposited into a computer available at Aklilu Lemma Institute of Pathobiology, Addis Ababa University.
